# Effectiveness of Artificial Intelligence for Personalized Medicine in Neoplasms: A Systematic Review

**DOI:** 10.1155/2022/7842566

**Published:** 2022-04-07

**Authors:** Sorayya Rezayi, Sharareh R Niakan Kalhori, Soheila Saeedi

**Affiliations:** ^1^Ph.D. Candidate in Medical Informatics, Health Information Management and Medical Informatics Department, School of Allied Medical Sciences, Tehran University of Medical Sciences, Tehran, Iran; ^2^Associate Professor, Health Information Management and Medical Informatics Department, School of Allied Medical Sciences, Tehran University of Medical Sciences, Tehran, Iran; ^3^Research Fellow, Peter L. Reichertz Institute for Medical Informatics, Technical University of Braunschweig and Hannover Medical School, 38106 Braunschweig, Germany

## Abstract

**Purpose:**

Artificial intelligence (AI) techniques are used in precision medicine to explore novel genotypes and phenotypes data. The main aims of precision medicine include early diagnosis, screening, and personalized treatment regime for a patient based on genetic-oriented features and characteristics. The main objective of this study was to review AI techniques and their effectiveness in neoplasm precision medicine.

**Materials and Methods:**

A comprehensive search was performed in Medline (through PubMed), Scopus, ISI Web of Science, IEEE Xplore, Embase, and Cochrane databases from inception to December 29, 2021, in order to identify the studies that used AI methods for cancer precision medicine and evaluate outcomes of the models.

**Results:**

Sixty-three studies were included in this systematic review. The main AI approaches in 17 papers (26.9%) were linear and nonlinear categories (random forest or decision trees), and in 21 citations, rule-based systems and deep learning models were used. Notably, 62% of the articles were done in the United States and China. R package was the most frequent software, and breast and lung cancer were the most selected neoplasms in the papers. Out of 63 papers, in 34 articles, genomic data like gene expression, somatic mutation data, phenotype data, and proteomics with drug-response which is functional data was used as input in AI methods; in 16 papers' (25.3%) drug response, functional data was utilized in personalization of treatment. The maximum values of the assessment indicators such as accuracy, sensitivity, specificity, precision, recall, and area under the curve (AUC) in included studies were 0.99, 1.00, 0.96, 0.98, 0.99, and 0.9929, respectively.

**Conclusion:**

The findings showed that in many cases, the use of artificial intelligence methods had effective application in personalized medicine.

## 1. Introduction

Cancer refers to a set of diseases in which some body's cells decide to divide continuously, and as a result, they spread into surrounding tissues (“National Cancer Institute”). Cancer is a genetic disease that changes genes' function and can control the way cells divide [1, 2]. In 2018, there were 24.5 million cancer cases (16.8 million without nonmelanoma skin cancer [NMSC]) and 9.6 million cancer deaths worldwide. Most of the disability-adjusted life-years (DALYs) caused by cancer lead to 97% of lives lost and only 3% of living with a disability. The leading cause of cancer deaths and DALYs for men is related to TBL (Tracheal, Bronchus, and Lung) cancer (1.3 million deaths and 28.4 million DALYs). However, the common leading cause of cancer death and DALYs for women is related to breast cancer (601.000 deaths and 17.4 million DALYs) [3]. Due to the growing number of cancer cases globally, timely detection and selection of the best treatment are considered key steps. Detection of cancer in the early stages can significantly increase the possibilities of successful treatment [4]. Early detection of cancer is greatly influenced by two factors of early diagnosis and screening [5]. In recent years, due to the importance of analyzing the genetic profile of people with cancer, the method of using extensive genomic data in a new field called precision medicine has been introduced [6]. With precision medicine and the progression of next-generation sequence (NGS), patients' genomic profiles can be used for disease diagnosis, risk prediction, and treatment of diseases [7]. Thus, the main aims of precision medicine include early diagnosis, screening, and personalized treatment regime for patient based on genetic-oriented features and characteristics [8].

Precision medicine for treating diseases considers various factors, which can be referred to the genome of individuals, lifestyle, environmental factors, and characteristics of patients [9]. Precision medicine allows clinicians to select more effective and accurate therapeutic and preventive approaches to a specific illness such as cancer. It can work in subgroups of patients based on their genetic make-up, environmental factors, and lifestyle [10]. In cancer genomics, the multiomics data, literature mining and analyzing, and genotype-phenotype data through genome-wide association studies (GWAS) have enriched artificial intelligence methods and solutions, and this has allowed health providers to give personalized care by precision medicine [11].

Artificial intelligence is a branch of computer science that makes intelligent machines that behave intelligently like humans. Intelligent systems can understand complex situations, simulate human thinking and reasoning, and solve complex problems. [12]. Recent advances in the field of AI and machine learning methods have enabled them to be used in biomedical sciences and health care [13]. AI uses a set of theories, algorithms, and computing powers to perform intelligent tasks such as decision-making, reasoning, language understanding, speech recognition, and visual perception [14]. AI can increase the speed of data analysis and accuracy of decision-making in the medical area [13]. Yet, using AI algorithms in precision medicine to predict, diagnose, and treat cancer is relatively new.

### 1.1. Objectives

The main objective of this study is to review the applications of AI algorithms and their effectiveness in personalized medicine approaches. This systematic review tries to respond to the main subsequent questions: RQ1: What are the applications of “AI neoplasms personalized medicine”? RQ2: Which AI techniques or intelligent methods have been applied in cancer precision medicine? RQ3: In which category do each of the AI approaches fall? RQ3: How successful AI methods have been reported to improve the care of neoplasms patients?

Performing this systematic review will give researchers a broad perspective on applying various artificial intelligence techniques in personalized medicine. Also, by examining the effectiveness of different artificial intelligence techniques, researchers can select techniques that have been highly accurate in personalized medicine. This study will also introduce software and data sources used in personalized medicine for cancer. They can also have a broad view of personalized medical applications in diagnosing and treating cancer.

## 2. Methods

The following Preferred Reporting Items for Systematic Reviews and Meta-analysis (PRISMA) for 2020 proposed by Page et al. were used in this study [15].

### 2.1. Eligibility Criteria

SPICE is a useful tool (like PICO) for asking focused clinical questions and qualitative reviews. The acronym SPICE stands for Setting, Perspective, Intervention, Comparison, and Evaluation and presents a way to formulate practice questions for finding evidence in existing research. SPICE may be more appropriate for formulating our research questions:

- Setting: All publications in the world
- Perspective: Patients and health providers
- Intervention: Artificial intelligence
- Comparison: Precision medicine
- Evaluation: What is the effectiveness of selected papers

Studies with the following inclusion and exclusion criteria were included in this review.

#### 2.1.1. Inclusion Criteria

The studies that met all the following criteria were entered in the review:

1. Original articles and proceedings
2. The system was designed for diagnosis, prediction, risk assessment, treatment, or screening of cancers
3. One of the AI methods was used for modeling
4. The diagnostic accuracy of the system was reported
5. The genomic, radiomic, proteomic, or phenotype data were applied in AI methods
6. Articles with English language
7. Papers that examined human-related neoplasms
8. All related studies without time limitation

#### 2.1.2. Exclusion Criteria

The exclusion criteria were as follows:

- The results of system test were not reported quantitatively
- Other than journal articles and proceedings such as review papers, letters, and book chapters
- The papers whose English full text of them was not available
- The studies whose knowledge modeling approach was not explicitly explained

### 2.2. Information Sources and Search Strategy

A systematic search was conducted in electronic databases including Web of Science, Medline (through PubMed), Scopus, IEEE Xplore Digital Library, and Cochrane Central Register of Controlled Trials to identify relevant studies published inception to December 29, 2021; we did not set a time limit for retrieving articles. Also, we searched Embase database until January 10, 2020. The search strategy used in this study included a combination of keywords and Medical Subject Headings (Mesh) terms related to “neoplasm,” “precision medicine,” and “artificial intelligence.” Table 1 shows the complete list of keywords and terms used in the search strategy for Scopus database. A reference manager software (EndNote X8, Thomson Reuters) was utilized to collect references and to exclude duplicates. The dates of coverage for each database is given in Table 2.

### 2.3. Study Selection

**Figure 1:**
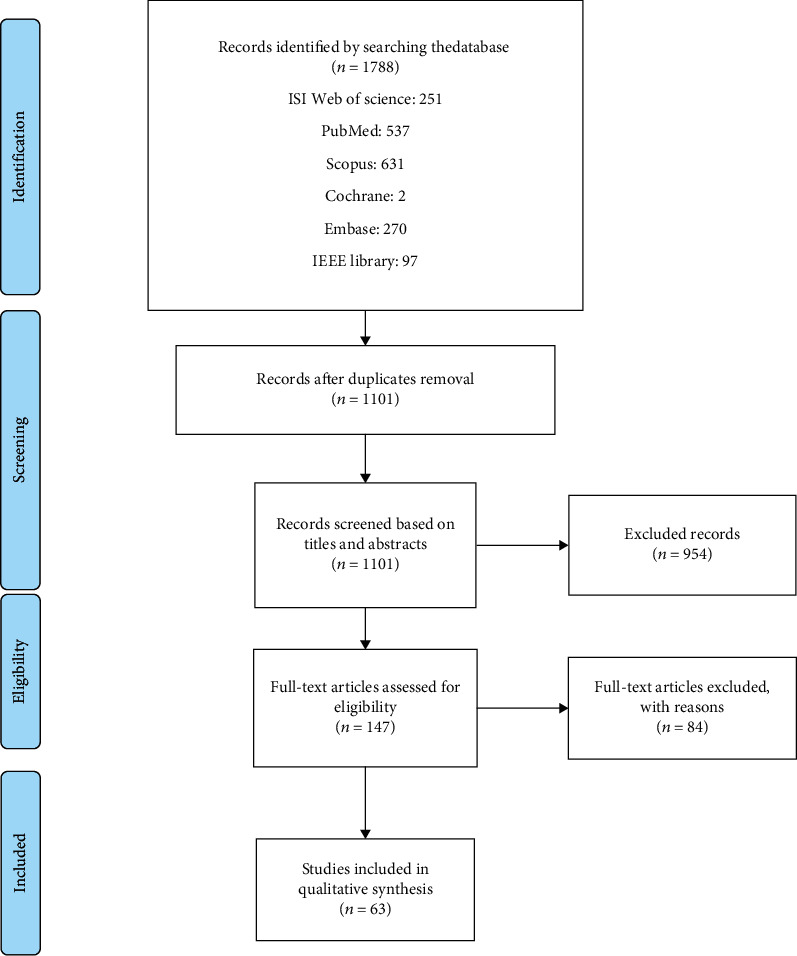
The flow diagram of identifying, selecting, and screening of papers based on PRISMA.

In this stage, assessment of records was done by more than one reviewer. The titles and abstracts of the identified articles were independently screened by two reviewers (S.R and S.S). The full text of the articles was retrieved and examined if it was supposed potentially relevant by two reviewers. Any disagreement between the reviewers was resolved by discussion by the third researcher. The following data were extracted from the selected studies and entered into a structured form in Excel. Data were extracted by each of the reviewers, and then, the forms were compared with each other. The screening procedures are displayed in Figure 1 based on the 2020 PRISMA method.

Meanwhile, the main classification of reviewed articles was determined by two authors independently. The two authors (S.R and S.S) analyzed and synthesized the main characteristics of selected papers, and then, they extracted the main specification of papers. The next author (S.RNK) evaluated the extracted information and validated the main elements.

### 2.4. Data Collection Process and Data Items

**Figure 2:**
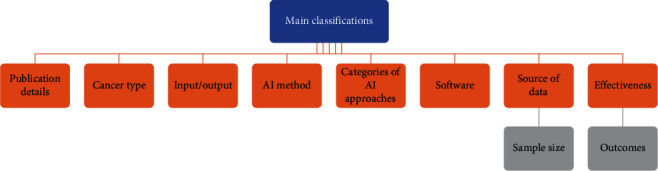
The main specifications of selected papers.

The first reviewer (SR) gathered the required information from the selected studies. Then, a second reviewer (SS) verified the accuracy of the information accumulated. Any dissensions were examined and resolved with a third reviewer (S.RNK). The main data elements and specifications of selected papers are displayed in Figure 2.

### 2.5. Study Risk of Bias Assessment

The Joanna Briggs Institute (JBI) critical appraisal checklist for analytical cross-sectional studies was used to assess the risk of bias of studies. The purpose of this appraisal is to assess the methodological quality of studies and has eight questions in the following order:

1. Were the criteria for inclusion in the sample clearly defined
2. Were the study subjects and the setting described in detail
3. Was the exposure measured in a valid and reliable way
4. Were objective, standard criteria used for measurement of the condition
5. Were confounding factors identified
6. Were strategies to deal with confounding factors stated
7. Were the outcomes measured in a valid and reliable way
8. Was appropriate statistical analysis used

These questions can be answered with four options: (1) yes; (2) no; (3) unclear; and (4) not applicable.

Each “yes” answer corresponds to one score, and if 70% of the questions answered “yes” in a study, the risk of bias was considered “low.” If 50% -69% of the questions were answered yes, the risk of bias was considered “moderate,” and below 50% considered “high risk [16].” The checklist was completed by two authors (SR and SS), and in case of disagreement between the two authors, the disagreement was resolved through discussion with the third author (S.RNK).

### 2.6. Data Synthesis and Analyses

In our review, meta-analysis was not performed as the methodology and methods of reporting results in included studies were heterogeneous. The results of selected studies had been reported by descriptive statistics.

### 2.7. Sensitivity Analyses Conducted to Assess Robustness of the Synthesized Results

We reviewed studies on the effectiveness of artificial intelligence techniques in personalized medicine, and the performance of which must have been quantitatively expressed. Studies with a low risk of bias were also included in the analysis. Studies should also have used cancer data to evaluate performance.

### 2.8. Assess Risk of Bias due to Missing Results in a Synthesis

In this study, we had no missing results and no risk of bias due to missing results.

### 2.9. Processes Used to Decide which Studies Were Eligible for each Synthesis

In this systematic review, we compared and synthesized the results of studies in which the performance of artificial intelligence techniques was presented quantitatively and in the form of accuracy, precision, recall, specificity, sensitivity, AUC (area under the ROC-curve), F-score, positive predictive value, negative predictive value, Mean Average Error (MAE), and Mean Square Error (MSE). Also, due to the complexity and a large number of types of artificial intelligence techniques, we classified them into several categories, which included the following: linear model, nonlinear model, rule-based system, NLP, deep neural network, neural network, and the Bayesian model. After classifying the types of artificial intelligence techniques in these categories, we performed the syntheses. The various beforementioned indicators are defined in the following as equations:

(1) $$\begin{array}{c} \text{Sensitivity and Recall}=\frac{\text{TP}}{\text{TP}+\text{FN}},\\ \text{Specificity}=\frac{\text{TN}}{\text{FP}+\text{TN}},\\ \text{Accuracy}=\frac{\text{TP}+\text{TN}}{\text{TP}+\text{TN}+\text{FP}+\text{FN}},\\ \text{Precision}=\frac{\text{TP}}{\text{TP}+\text{FP}},\\ \text{MAE}=\frac{\;\sum_{i=1}^{n}\;|\;yi-x\;i\;|\;}{n},\\ \text{F}-\text{score}=2\times \frac{Precision\times Recall}{Precision+Recall},\\ MSE=\frac{1}{n}\overset{n}{\underset{i=1}{\sum }}{\left(Y_{i}-{\overset{\wedge }{Y}}_{i}\right)}^{2}, \end{array}$$

where *y*_*i*_ = prediction, *x*_*i*_ = true value, and *n* = total number of data points.

Here, TP: true positive, TN: true negative, FP: false positive, and FN: false negative [17].

The ROC curve is constructed by plotting the true positive rate (TPR) versus the false positive rate (FPR) in diverse threshold sets. It is ideal for maximizing the TPR while minimizing the FPR. This means that the top left corner of the plot is the ideal point (FPR = 0 and TPR = 1).

## 3. Results

### 3.1. Study Selection

**Figure 3:**
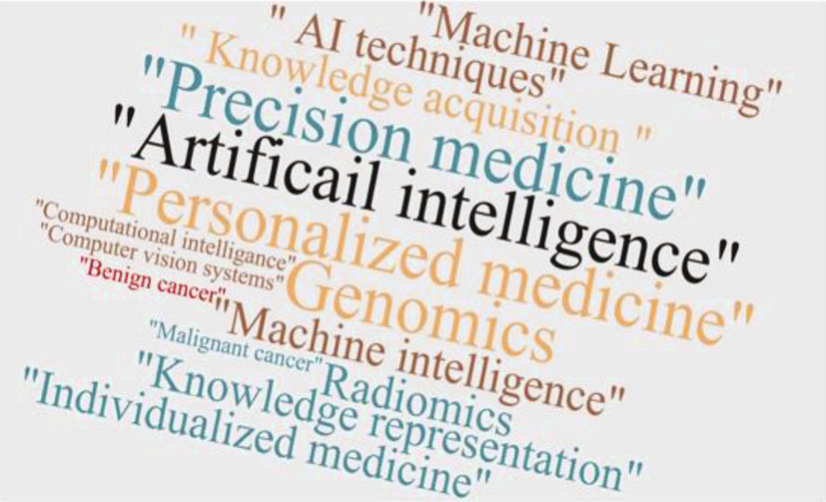
Word cloud of main keywords in selected papers.

A total of 1788 relevant articles resulted from the search until December 29, 2021. After removing the duplicates, 1101 articles remained. Hence, in the last phase, only 63 papers that met the inclusion criteria were reviewed. In Table 3, a summary of the main results and characteristics of the papers is illustrated. Outcome measurements including results and effectiveness are summarized in Table 4. The main keywords used for selecting the papers are displayed by a word cloud scheme.  Figure 3 presents the more weighted and frequent key terms used in the search. In this figure, the notion of keywords is demonstrated.

### 3.2. General Characteristics of the Included Studies

**Figure 4:**
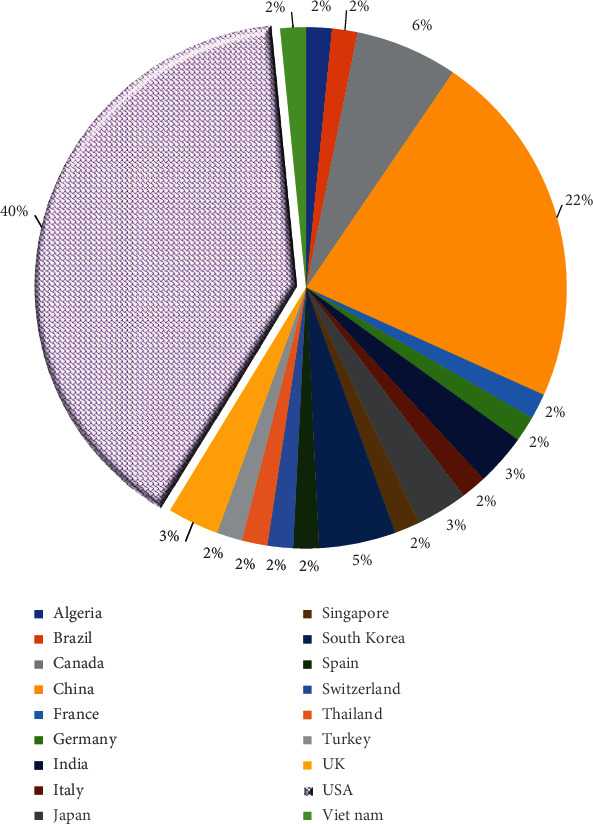
The distribution of articles based on countries.

**Figure 5:**
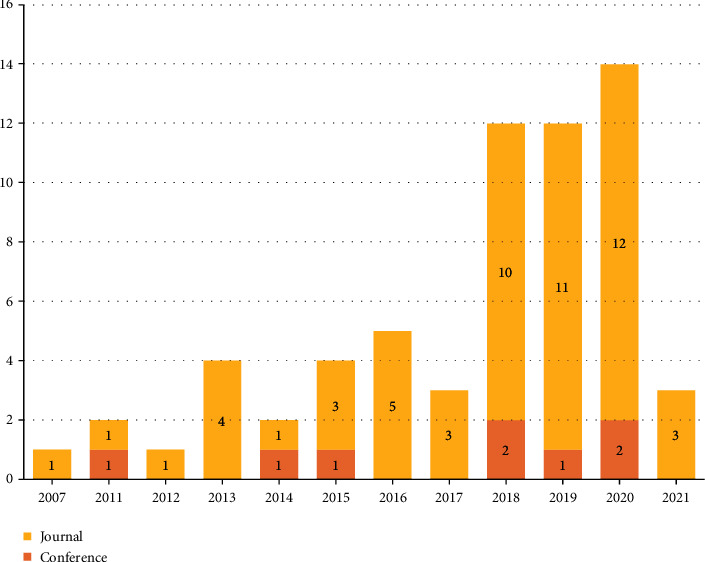
The distribution of papers based on publication year and type.

The reviewed papers are presented in Figure 4 based on publication country. Forty percent of all papers were conducted in the USA, and 22% in China. The other remained countries had a relatively equal number of published articles. *The frequency of selected articles based on their publication type for each year is displayed in*Figure 5*. The papers included in this review had been published between 2007 and 2021. As it is seen, a large number of papers had been published in 2020*, *2019*, *and 2018. Meanwhile, the articles published in conferences are less than these, which have been presented in different scientific journals.*

### 3.3. Source of Data and Sample Size

The selected papers had mostly publicly available data. These sources included gene expressions, gene sequencing data, phenotyping data, and somatic and mutation data. However, the molecular interactions, drug chemical data, radionics data, and pharmacogenomics data were stored in the sources. Out of 63 articles, in 19 papers, The Cancer Genome Atlas (TCGA) was used as the source of datasets. However, in 14 articles, the Cancer Cell Line Encyclopedia (CCLE) and Genomics of Drug Sensitivity in Cancer (GDSC) sources were employed as the source of datasets. In five papers, the required datasets were extracted from medical and electronic health records, and in six articles, the public websites and recruited data in papers were applied as the source of datasets. Ultimately, some other types of sources were applied in the remained papers. These sources are illustrated in detail in Table 3. Out of 63 papers, in 38 papers, the sample size was reported as patient samples, but in 16 reviewed papers, the sample size was reported as biosamples like genes, molecular samples, and cell lines. These types of samples have a large number of sizes. The reported sample size in 32 papers ranged from 30 individuals to 26,000, and also in some papers, the sample size was not mentioned.

### 3.4. The Distribution of Selected Papers Based on Applied Software

Out of 63 citations, in 21 articles (33.3%), the software or technical environment was not reported, whereas, in 12 papers (19.4%), the popular and frequently used software was the R package. However, in nine of the reviewed articles, MATLAB has been used alone or in combination with other software for analysis. Also, in 10 citations (15.87%), the popular software used in the study included Weka, Python libraries, and Tensor Flow. In the other reviewed articles, software such as IBM Watson for Genomics version 27.87, PLATYPUS version 1.0, Proteomics Performance Evaluation Pipeline Software (PSPEP), and graph visualization software were used.

### 3.5. The Characteristics of Selected Articles Based on Input, Cancer Types

**Figure 6:**
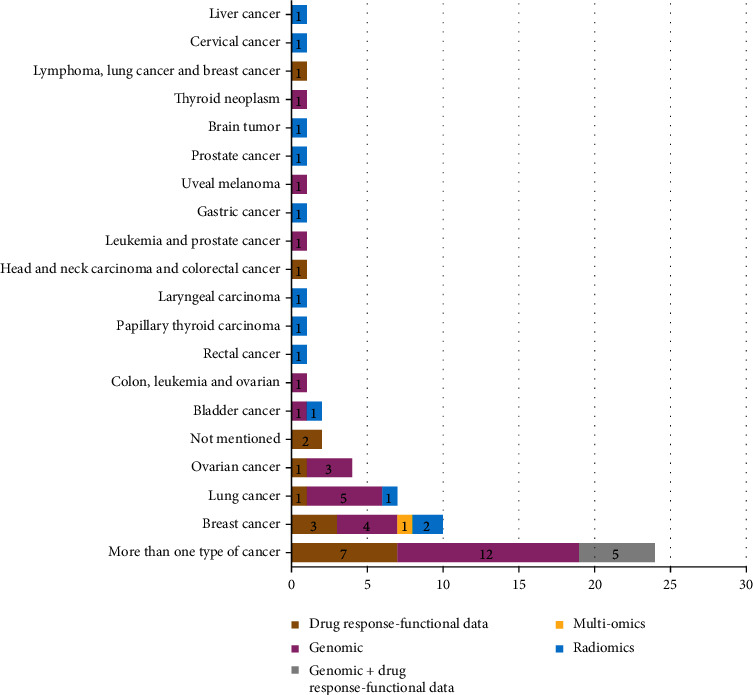
The distribution of papers based on inputs and type of cancer.

**Figure 7:**
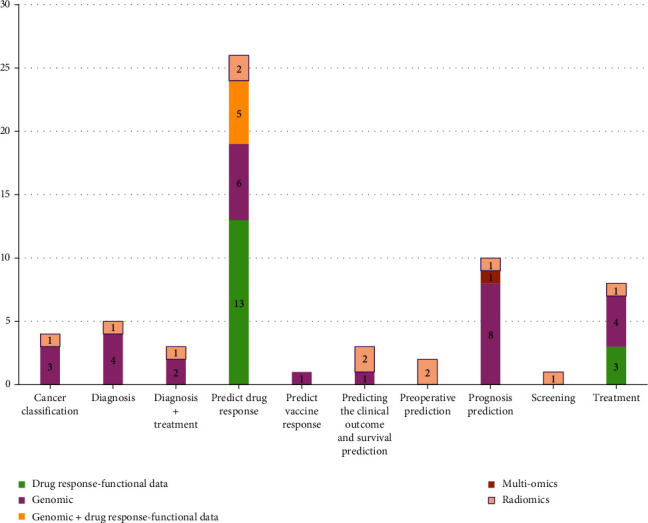
The distribution of citation by inputs and type of care.

First of all, it can be said that the inputs of applied AI methods and algorithms were categorized into genomic data (gene expression, somatic mutation, phenotype data, sequencing data, and proteomic data), functional data, and radiomics data (radiogenomic biomarkers and histology of images). Based on literatures, the genomic data include profile of DNA, proteomics, transcriptomics measure transcripts, metabolomics, and radiomics. These concepts have created multiomics new profiles. Radiomics is an approach that extracts a large number of features and critical characteristics from radiographic images. Hence, detecting correlations with genome patterns is mining if radiomic data is known as radiogenomics (59, 60). Out of 63 papers, in 34 articles (53.9%), genomic data like gene expression, somatic mutation data, phenotype data, proteomics with drug-response data was used as input in AI methods; in 12 citations (19.4%), radiomic data (radiographic with biomarkers) was applied by researchers for managing neoplasm' treatments. However, in 16 papers (25.3%) drug response, functional data was applied in personalization of treatment. drug response which is functional data. Concerning the type of cancer, in seven papers out of 63, the selected cancer for treatment (predicting, diagnosing, and treating) was breast cancer, and the data of lung cancer was used in seven reviewed papers. Also, in four citations, the data of ovarian cancer was used by the researchers. In some papers, the data of cancers such as bladder cancer, thyroid cancer, colorectal cancer, brain tumor, laryngeal carcinoma, leukemia, and neck cancer were utilized too. The type of selected cancers and type of inputs (source of omics data) are displayed in detail in Figure 6. However, in Figure 7, the distribution of papers by the type of care and input is depicted.

### 3.6. Distribution of Selected Papers by Effectiveness

The effectiveness of selected AI methods in various reviewed papers is shown in Table 5. The results showed that AI algorithms have the potential and capacity to predict, diagnose, and treat cancer (drug-chemotherapy evacuation, etc.). These methods can classify or stage patients and provide better therapy measures. The performance of applied methods was validated and evaluated by different beforementioned criteria, including accuracy, sensitivity, specificity, AUC of ROC, Mean Average Error (MAE), and F-measure. Maximum values of the assessment indicators such as accuracy, sensitivity, specificity, precision, recall, AUC, F-score were calculated to be 0.99, 1.00, 0.96, 0.98, 0.99, and 0.9929, 0.98, respectively. These reported criteria showed that the performance of the methods was at a significant level. Hence, many of the algorithms proposed in reviewed studies have effectively performed early detection of cancers, predicting response to treatment, and screening through personalized medicine.

### 3.7. The Distribution of Citations Based on the Type of Presented Care by AI Methods

**Figure 8:**
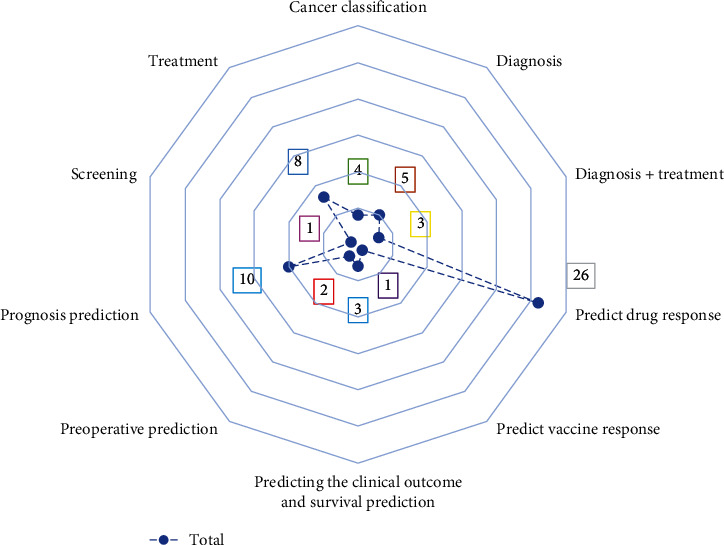
The distribution of papers based on the type of care.

Based on extracted results, in 41% of studies, the main purpose of using genomic data in artificial intelligence was to predict the response to drugs in the treatment of cancer patients and in 12.7% of the papers, correct diagnosing of neoplasms by AI approaches was the critical care. The offered type of care in studies is displayed in Figure 8.

### 3.8. The Distribution of Artificial Intelligence Methods in Selected Papers

The main objective of this review was to determine the application of AI techniques in precision medicine for cancer screening, diagnosis, and treatment. In Table 4, an overview of the distribution of applied AI algorithms, their categorizations in the selected papers, and the frequent methods used in the reviewed papers are presented. Out of 63 citations, the leading AI approaches in 17 papers (26.9%) include linear and nonlinear models (classification and regression trees, support vector machine, Neural Networks, and etc.). In 15 articles (23.8%), unique methods based on artificial intelligence were used, considered linear models (Random Forest or Decision Trees). Totally, in 21 citations, rule-based systems and deep learning models were used too. Some other intelligent techniques such as metaensemble feature selection, kernel learning, natural language processing, and the least absolute shrinkage and selection operator Cox regression were employed by researchers to determine cancer characteristics and input methods.

### 3.9. Risk of Bias within Studies

Sixty included studies in this review were considered low risk of bias. Only two citations were evaluated with moderate risk of bias [18, 19] and one with high risk of bias [20]. The questions “Were confounding factors identified? and “Were strategies to deal with confounding factors" are not applicable in our included studies, because our studies were not experimental researches.

## 4. Discussion

According to the results, the leading artificial intelligence methods and applications are widespread and lead to knowledge-based production or model development applied widely in healthcare fields. The main objective of this review was to analyze and identify the studies conducted on the application of AI methods in precision or personalized medicine for cancer prediction, diagnosis, and treatment. To achieve this objective, we selected 63 papers based on inclusion and exclusion criteria. The basic aim of reviewed studies was to provide or propose AI-based approaches that can predict the outcomes of treatments such as drug therapy or chemotherapy and patient screening/diagnosis. However, it should be noted that the type of care in a large number of selected citations was predicting the type of treatment based on the stage of cancer and identifying the mutations and sequencing in genomics.

Support vector machine is one of the most preferred methods of machine learning that has high accuracy. This algorithm is capable of handling a large volume of data [21], and it is also a method of choice when dealing with large and complex data as it can provide statistical analysis and summarization [22]. Another algorithm employed in the reviewed papers was the random forest algorithm, which can be used widely in more applications, specifically with large datasets.

Hence, based on effectiveness reports, in large numbers of the selected papers, the SVM-based and RF methods effectively predicted and diagnosed cancer with genomic data. Another algorithm that was used in radiomic-oriented papers was convolution neural network (CNN), which is a deep learning technique that can take in an input image and is designed to improve the accuracy of automatic labeling and classification [23, 24]. In some reviewed papers, the researchers used CNN to propose and predict cancer patients based on radiogenomics and histology images. Based on our results, the qualitative criteria such as ROC, accuracy, sensitivity, specificity, precision, and F-score were reported separately for reviewed papers. For example, in a study in 2019 that examined the response of drug and chemotherapy in patients with cervical cancer, the sensitivity of SVM models was higher than 84% and AUC was 0.99 for testing the set [25]. In 2018, Brietenstein et al. proposed a rule-based algorithm for diagnosing and treating patients with breast cancer, which had the precision of 0.98, recall of 0.99, and F-score of 0.98 [26]. Meanwhile, Chen et al. proposed a radiomic-based model for selecting proper surgical approaches in papillary thyroid carcinoma; this model had an AUC of 0.837 and F-score of 0.766 [27]. In another work, an SVM-based classification model was proposed for cancer prediction, which had an accuracy of 70% and AUC of 0.9929 [28]. Similarly, Kempowsky-Hamon et al. developed a fuzzy logic algorithm for predicting breast cancer prognosis; the performance of this model was reported by sensitivity and specificity (0.95 and 0.93, respectively) [29]. Notably, in a study which is conducted in 2020, a novel technique was developed as a result of comparisons providing practical guidance on selecting machine learning workflows and their tuning to generate well-calibrated CP estimates for precision diagnostics operating DNA methylation data; the highest AUC of RF, ELNET, and SVM, are 99.9%, 99.8%, and 85.0%, respectively [30]. In [31], the consequences demonstrate offered ensemble transfer learning methods sweeten the prediction performance in all three drug response prediction applications with all three prediction algorithms. According to the drug response data, AUC values (0.98, 0.99) are computed and abode as the drug response measurements to be predicted through regression analysis. Nevertheless, due to the heterogeneity of reports and our results, we could not analyze them one by one. However, the selected papers showed that their methods were influential in the precision medicine field.

Our results showed that the main type of cancer was breast and lung cancer in most of the selected studies. Breast cancer is cancer that forms in the cells of the breasts. After skin cancer, breast cancer is the most common cancer in women. Based on results, breast cancer survival rates have increased, but the number of death associated with this cancer is declining [32]. However, breast cancer is the most invasive cancer in women and the second cause of death after lung cancer [6, 7]. For this reason, a new personalized approach was introduced with the name of precision medicine used to diagnose, treat, and prevent the number of cancers such as breast cancer by taking into account the genes (genetic makeup) or other markers in the cancer cells [33]. In other words, if the cancer treatment and diagnosis in individuals are based on the formation of genome profiles, personalized medicine is a more effective method that can be used in the treatment process [34]. The blood or tumor tissue in this method is collected for genetic analysis to determine its genetic makeup, which may later help predict or diagnose cancer or guide the treatment decisions. Other tests determine the genetic changes or variants (called mutations) within the cancer cells. This information can help determine which treatments are most likely to be beneficial or if any treatment is needed at all. For example, cells from a breast tumor may be tested to determine whether they produce too much of a protein called HER2 [35]. Lung cancers differ according to the type of cell in which they arise. In these cancers, specific molecular targets have been identified, and which gene alterations produce mutations [36]. Therefore, if the genetic abnormality is identified, it can be targeted by a drug [37]. According to our results, *genetic is very effective in the onset of breast and lung cancer, which were the most common types of cancer in the selected papers. The source of our reviewed papers was publicly available. These sources include gene expressions, gene sequencing data, phenotyping data, and somatic and mutation data. However, the molecular interactions and drug chemical data, radiomics data, and pharmacogenomics data were stored in the sources. In most of the selected papers, TCGA, CCLE, and GDSC sources were used. These three sources have the main datasets on patients or biosamples, and their data are publicly available with various types of genomics and radiomics data.*

The results showed that most of the articles had been published in the United States, and the number of articles published in the field of precision medicine has increased significantly in recent years. Various factors may have led to an increase in publications of such articles in recent years, especially in the United States. The term “precision medicine” was first highlighted in a publication by the US National Research Council, which sought to create a new taxonomy for classifying diseases through a knowledge network [38, 39]. US National Human Genome Research Institute (NHGRI) has developed a 20-year plan for translating insights from genomics to medicine. This has led to an understanding of human biology and disease prevention, diagnosis, and treatment [39, 40]. President Obama, on January 20, 2015, announced that “Tonight, I am launching a new Precision Medicine Initiative to bring us closer to curing diseases like cancer and diabetes—and to give all of us access to the personalized information we need to keep ourselves and our families healthier.” This could have led to progress in precision medicine [41]. President Obama has allocated $215 million for the initial launch of this initiative. He also donated $130 million to the Cohort study, which involved at least one million volunteers. This can lead to the collection of genotypic, phenotypic, and lifestyle data and thus accelerate the development in this scientific field [42].

All of the mentioned items can be the reason for the advancement of precision medicine in the United States, especially in recent years. It seems that other countries should take a step in this direction and keep pace with the United States in developing this area by allocating sufficient budget and time.

This study had several strengths. One of the strengths of this study was searching in critical databases including Medline (through PubMed), Scopus, Cochrane Central Register of Controlled Trials, Embase, IEEE Xplore Digital Library, and ISI Web of Science, which enabled us to cover all the articles published in this field as much as possible. Another strength of this study was the inclusion of papers presented at the conferences. We also did not impose any time limit on the search.

In this study, we faced some limitations, one of which was the noninclusion of studies presented at conferences, that we did not have access to their full texts. We also used only English papers, so there is the probability of missing several related studies and papers with effective results. However, we could not update Embase search due to lack of access to Embase database from our country.

## 5. Conclusion

This review was conducted to examine the applications of artificial intelligence in personalized medicine. To achieve this goal, we investigated five important databases to retrieve published scientific papers without time limitation. Hence, applying appropriate AI-based solutions could improve the treatment and management of cancers and the application of intelligent approaches is recommended in many areas such as in personalized medicine. However, further studies are needed to investigate the real effects of these algorithms and their effectiveness.

## Figures and Tables

**Table 1:** Applied search strategy for Scopus database.

Scopus	“TITITLE-ABS-KEY ((“Neoplasia” OR “Neoplasia” OR “Neoplasm” OR “Tumors” OR “Tumor” OR “Cancer” OR “Cancers” OR “Malignancy” OR “Malignancies” OR “Malignant Neoplasms” OR “Malignant Neoplasm” OR “Neoplasm, Malignant” OR “Neoplasms, Malignant” OR “Benign Neoplasms” OR “Neoplasms, Benign” OR “Benign Neoplasm” OR “Neoplasm Benign”) AND (“Artificial Intelligence” OR “Computational intelligence” OR “machine intelligence” OR “Computer vision systems” OR “Knowledge acquisition “OR “Knowledge representation “OR “Computer reasoning”) AND (“Precision Medicine” OR “Personalized medicine” OR “ Individualized medicine”))

**Table 2:** The dates of coverage for each database.

Database	Coverage
Scopus	2007-2021
PubMed	2008-2021
Embase	Until 2020
ISI Web of Science	2014-2021
IEEE Xplore	2007-2021
Cochrane	2017-2021

**Table 3:** The characteristics of reviewed papers entered in the study based on inclusion criteria.

#	Author	Journal/ Conference name	Research aim	Cancer Type	Kind of care (Prediction, Screening, Diagnosis, Treatment)	AI method	Categories of AI approaches	Input	Output	Software	Source of Data set	Sample Size #
1	Le and Pham [43], Vietnam	Journal of Molecular Biology	The main aim of this work was developing a novel network-based method, named GloNetDRP, to overcome response limitation	More than one type of cancer	Predict drug response	GloNetDRP	Linear model	Genomic	Cell line similarity and drug similarity for predict response of to drug	Not mentioned	CCLE + GDSC	Not mentioned

2	Gatta et al. [44], Italy	Artificial Intelligence in Medicine	The objective of this paper was introducing a medical agent-based decision support system capable of handling the whole radiomics process and return a prediction about the clinical outcome of a proposed treatment	Rectal cancer	Prediction of cancer from samples	LR+ FE	Rule-based system	Radiomics	Predicting the outcome of a previously unseen clinical case	RadAgent software	To provide the appropriate input for the proposed approach, MR scans have been processed using the moddicom R library	Data set 1: 173 patients Data set 2: 25 patients

3	Shimizu and Nakayama [45], Japan	EBioMedicine	The aim of this work was developing a new prognostic score based on intelligence models (random forest and neural network) that is enforceable to a wide range of patients with breast cancer	Breast cancer	Prognosis prediction and scoring stage	RF+ NN	Linear and nonlinear models	Genomic	Overall Survival (OS) or clinical stage and disease-free survival (DFS)	Not mentioned	TCGA + molecular taxonomy of Breast Cancer International Consortium	Patients: 11,893

4	Sun et al. [25] China	EBioMedicine	The main aim was constructing a predictive model for predicting the response to neoadjuvant chemotherapy (NACT) by radiomic analysis	Cervical cancer	Predict drug response	SVM	Linear model	Radiomics	“responders” (chemosensitive); “nonresponders” (chemoinsensitive)	MATLAB	No public data: Nanfang hospital	Total 275 patients (183 patients training sets; 92 patients testing sets)
5	Overby et al. [46], USA	The Journal of the American Medical Informatics Association	This study was presenting a knowledge-based approach to derive “phenotype score” based on Pharmacogenomics knowledge. This model has prediction power for metabolic process in drug treatments (to breast cancer patients taking tamoxifen	Breast cancer	Predict drug response	PEMRIC	Rule-based system	Drug response-Functional data	Endoxifen/ NDM plasma levels	Not mentioned	Clinical data source; Pharmacogenomics Knowledge Base and evidence-Base (PharmGKB, SuperCYP)	Patients: 30

6	Urbanowicz et al. [47], USA	The Journal of the American Medical Informatics Association	The aim of this work was detecting complex patterns of association between genetic on environmental risk factors	Bladder cancer	Diagnosis	AF-UCS	Non-linear model	Genomic	Tumor stage and grade, age at diagnosis (years), survival time in years	Graph visualization software	Source of data set was provided by Andrews et al. work.	Case group: 355 control group: 559

7	Ding MQ et al. [48], USA	Molecular Cancer Research	The aim of this study was to identify drugs that will be effective treat neoplasms	More than one type of cancer	Predict drug response	SVM	Linear model	Drug response-Functional data	The name of targeted drugs and percentage of cell line responsive	Not mentioned	GDSC + CCLE	The 624 cell lines were randomly split into training and testing data sets of 520 and 104 samples,

8	Diggans et al. [49], USA	Pacific Symposium on Biocomputing	In this article, the main aim was developing a machine learning approach for identifying the BRAF V600E mutations using MRNA expressions in thyroid fine needle aspirate biopsies (FNABs)	Thyroid neoplasm	Diagnosis	SVM	Linear model	Genomic	Malignant nodules and benign nodules	Not mentioned	Not mentioned	Patients: 716
9	Gligorijevic et al. [50], UK	Pacific Symposium on Biocomputing	In this work, the main aim was introducing a versatile data fusion framework that was based on graph-regularized nonnegative matrix trifactorization, a machine learning technique for co-clustering heterogeneous datasets	More than one type of cancer	Predict drug response	NMTF	Linear model	Genomic	Patient subgroups (stratification) with prognostic survival outcome, predicting novel driver genes and repurposing drugs (predicting new candidate drugs)	Not mentioned	TCGA4 with molecular networks (MNs) from BioGRID17 and KEGG18, drug-target interaction (DTI) and DrugBank	Patients: 353

10	Acar et al. [51], Turkey	The British Journal of Radiology	In this study, the team used different machine learning methods for distinguish the lesions images	Prostate cancer	Treatment	DT+ SVM+ KNN+EC	Linear and non-linear models	Radiomics	Metastasis/Completely responded lesions	LifeX software-MATLAB software	Medical records	Patients: 75

11	Alaa AM et al. [52], USA	IEEE Transactions on multimedia	The objective of this work was presenting a CDSS for stratifying cluster of patients	Breast cancer	Screening	ConfidentCare	Rule-based system	Radiomics	Recommend a regular (1 year) follow-up, recommend a diagnostic test (biopsy)	Not mentioned	EHR data in the United States	Patients: 25,594

12	Boucheham and Batouche [53], Algeria	Science and Information Conference	The main aim was proposing a novel algorithm for biomarker discovery in cancer diagnosing	Colon, leukemia and, ovarian	Diagnosis	MEFS	Linear model	Genomic	The best selected features	MATLAB	Kent Ridge biomedical data repository	Patients with colon cancers: 62 leukemia: 72 ovarian cancers: 253

13	Breitenstein et al. [26], USA	Clinical and Translational Science	The main aim was proposing a rule-based algorithm which could create robust precision medicine phenotypes in breast cancer patients from HER perspectives	Breast cancer	Diagnosis + treatment	NLP	NLP	Genomic	Receptor status phenotypes: overexpression in BC patients	Not mentioned	Cancer registry data + EHR data.	Patients: 13,162
14	Nikolova et al. [54], USA	Bioinformatics	The aim was proposing a novel, biologically motivated, Bayesian multitask approach, which explicitly models gene-centric dependencies across multiple and distinct genomic platforms for the identification of drug response biomarkers	More than one type of cancer	Predict drug response	GBGFA	Bayesian model	Drug response-functional data	Drug recommend based on cell lines patients	Not mentioned	CCLE + CTRP + TCGA PAAD+ LUAD cohorts	Biosamples: 267 cell lines + 409 cell lines patients: 132+ 165

15	He et al. [55], Switzerland	Bioinformatics	The aim of this work was proposing a ML approach which named Kernelized Rank Learning. This method ranks drugs based on patient's molecular profile	Breast cancer	Treatment	KRL	Linear and nonlinear models	Drug response-functional data	Drug recommend based on cell lines patients	Python + MATLAB	GDSC + TCGA	Not mentioned

16	Mobadersany et al. [56] USA	Proceedings of the National Academy of Sciences of the United States of America	The aim of this work was developing a computational method based on deep learning for predicting the outcome of patients with brain tumor	Brain tumor	Predicting the clinical outcome and survival prediction	CNN	Deep learning model	Radiomics	Grading diffuse gliomas and suggest relevance for patterns with prognostic significance	TensorFlow	TCGA Lower-Grade Glioma (LGG) and Glioblastoma (GBM) projects.	Patients: 769

17	Fathiamini et al. [57], USA	Journal of American Medical Informatics Association	The objective of this work was creating an automated system to identify drugs for cancer related genes in relevant literature	More than one type of cancer	Predict drug response	NLP	NLP	Drug response-Functional data	Detect relation between gene-drugs for treating cancers in clinical trials.	Not mentioned	SemMedDB: SemRep_UTH to process MEDLINE and ClinicalTrials.gov	183 260 trials (entire set) + 23 537 576 PubMed abstracts
18	Itahashi et al. [58], Japan	Frontiers in Medicine	The basic objective of this work was to assess the validity and utility of WfG for analyzing clinical genome sequencing results by comparisons with results obtained by an expert panel composed of multidisciplinary specialists at NCCH	More than one type of cancer	Diagnosis + treatment	WfG	Rule-based system	Genomic	Actionable or alterations with therapies	IBM Watson for Genomics	Hospital (TOP-GEAR PROJECT)	Patients: 198

19	Chen et al. [27] China	Frontiers in Medicine	The main objective was exploring a radiomic model for preoperative prediction of ETE in patients with PTC	Papillary thyroid carcinoma	Preoperative prediction	LR+ RF+ SVM	Linear and nonlinear models	Radiomics	Preoperative prediction of ETE	MATLAB	Dataset was recruited for patients in cohort study.	Patients: 624

20	Wang et al. [59], China	Frontiers in Medicine	The main objective was exploring and developing a new approach based on eight radiomic features for identifying the individuals' accurate preoperative T category for patients with advanced malignant laryngeal carcinoma	Laryngeal carcinoma	Preoperative prediction	SVM	Linear model	Radiomics	Preoperative T category (T3 vs. T4) for patients with advanced laryngeal cancer before surgery	Pyradiomics, Python + R software	Medical records	Patients: 211

21	Graim et al. [60], USA	Pacific Symposium on Biocomputing	The main aim of this work was proposing a multiple-view learning predictive framework for identifying the cancer drug sensitivity	More than one type of cancer	Predict drug response	MVL	Rule-based system	Drug response-functional data	Predicting drug sensitivity in cell lines	PLATYPUS	CCLE	Biosamples:1,037
22	Jones et al. [61], USA	BMC Medical Genomics	The main aim of this work was identifying reliable gene expression pattern for classifying tumor class using a local minimax kernel algorithm	Leukemia and prostate cancer	Prognosis prediction	KRL	Linear and nonlinear models	Genomic	Predicting the probability of malignancy with a level of confidence-diagnose	Not mentioned	Three publicly available gene expression datasets: data was extracted from papers	Tumor samples: 365 normal samples: 265

23	Kim et al., USA	IEEE International Conference on Bioinformatics and Biomedicine (BIBM)	The main aim of this work was proposing a framework based on personalized medicine with Reverse-Phase Protein Array (RPPA) and sensitivity of drugs	Lung cancer	Predict drug response	NB	Bayesian model	Genomic	High probability of low sensitivity or low probability of low sensitivity	Weka	Not mentioned	Biosamples: 55 antibodies and 75 lung cancer cells lines, cell lines per drug is 43

24	Kureshi et al. [62], Canada	IEEE Journal of Biomedical and Health Informatics	The main objective of this work was investigating the influence of a combination of factors-clinical predicators, environmental risk factors, and EGFR mutation. These can be used to predict the tumor response to EGFR-TKI therapy for patients with advanced-stage NSCLC	Lung cancer	Treatment	SVM + DT	Linear and nonlinear models	Genomic	Responder and nonresponder group (response to EGFR-TKI therapy.)	Weka	PubMed papers	Train set: 291 patients Test set: 64 patients

25	Li et al. [63], China	IEEE International Conference on Bioinformatics and Biomedicine (BIBM)	The main objective was developing a centric radiogenomics framework based on a deep learning approach for mapping the image features and characteristics and gene expression profile data	Lung cancer	Diagnosis	CNN	Deep learning model	Radiomics	Image features and patients' metagene: typical CT TR and gen information	Not mentioned	Dataset lung3 and NSCLC Radiogenomics are from cancer archive	Patients: 300
26	Lin et al. [64], China	European Radiology	The aim of this work was to develop a radiomics and genomic signature to predict clinical outcomes and prognosis of BLCA patients	Bladder cancer	Predicting the clinical outcome and survival prediction	LASSO	Nonlinear model	Radiomics	Prognostic indicators: High-risk or Low-risk	R software + ultrosomics software	TCGA + TCIA	Patients: 62

27	Menden et al. [65], UK	PLoS ONE	The main objective of this paper was to develop a machine learning model to predict sensitivity and drug responses based on genomic features and alterations	More than one type of cancer	Predict drug response	MLP + RF	Linear and nonlinear models	Drug response-Functional data	Cell survival and drug response	Encog + R + PaDELe	Genomics of Drug Sensitivity in cancer project	Cell line samples: 608 drugs:111

28	Moon et al. [66], USA	Artificial Intelligence in Medicine	The main objective of this work was proposing a new ensemble-based classification method than can be used to predict more effective therapies in patients for individualizing treatments	Lymphoma, lung cancer, and breast cancer	Predict drug response	CART	Linear model	Drug response-functional data	Classifying patients and drug response	R package	Public websites	Patients: 306

29	Assawamakin et al. [67], Thailand	BioMed Research International	The basic aim of this paper was developing a novel two-step machine learning framework which can present to address the prediction of phenotypic outcomes.	More than one type of cancer	Prognosis prediction	NB + HNB	Bayesian model	Genomic	Prediction of phenotypic and proteomic outcomes	Weka	KRBDSR + GEMLeR + NCICPD	Patients: 230

30	Majumder et al. [68], India	Nature Communications	The aim of this work was proposing a machine learning algorithm to predicting clinical response to anticancer drugs for engineering of personalized tumor ecosystems	Head and neck carcinoma and colorectal cancer	Predict drug response	SVM	Linear model	Drug response-Functional data	Ranking patients as CR (complete response), PR (partial response) or NR (nonresponse): These ranking had different drug regimens D1,D2, D3 or D4.	PSPEP Software	Dataset was recruited by project team.	Patients: 164
31	Sun et al. [69], China	Cancers	The main objective of this study was proposing artificial intelligence approach that could predict assessments of the level of BAP1 expression in enucleated eyes with unveil melanoma	Uveal melanoma	Cancer classification	DenseNet-121	Deep learning model	Genomic	Prediction of BAP1 classification: Yellow areas correspond to BAP1-classification “high” and green to “low”.	PyTorch toolkit + Python	Published papers	Patients: 47

32	Potie et al. [70], Spain	2019 IEEE International Conference on Fuzzy Systems (FUZZ-IEEE)	The objective of this study was to show the benefits of one of the learning paradigms of Computational Intelligence	Lung cancer	Cancer classification	Fuzzy model	Deep learning model	Genomic	Lung cancer prediction from samples taken by liquid biopsy.	KEEL + scikit-learn	GEO	Patients:779

33	Yu et al. [71], USA	Journal of Proteome Research	The objective of this work was predicting individual platinum response using robust machine learning models, and discovered proteins and biological processes associated with platinum response	Ovarian cancer	Treatment	RF+ SVM+NB	Bayesian model + Linear and nonlinear models	Genomic	Patient's response to platinum drugs	R package	TCGA + CPTAC	Patients: 130

34	Kalari et al. [72], USA	JCO Clinical Cancer Informatics	We propose a precision medicine computational framework, PANOPLY (Precision Cancer Genomic Report: Single Sample Inventory), to identify and prioritize drug targets and cancer therapy regimens	Breast cancer	Treatment	RF	Linear and nonlinear models	Drug response-Functional data	Personalized list of prioritized drugs	R package	Breast Cancer Genome Guided Therapy Study (BEAUTY)	Patients: 17
35	Klein et al. [73], USA	BMC Bioinformatics	The aim of this study was presenting GRAPE as a novel method to identify abnormal pathways in individual samples that is robust to platform/batch effects in gene expression profiles generated by multiple platforms	Breast cancer	Diagnosis	RF + SVM	Linear and nonlinear models	Genomic	Use healthy reference samples to quantify the abnormality of individual pathological samples.	R package	TCGA	Different sample size mentioned for different pathways

36	Dong et al. [74], China	BMC Cancer	The aim was the generation of genetic predictions of drug response in the preclinical setting and their incorporation into cancer clinical trial design	Not mentioned	Predict drug response	SVM	Linear model	Drug response-Functional data	Response to anticancer drugs	R package	CCLE + CGP	Not mentioned

37	Kempowsky-Hamon et al. [29], France	BMC Medical Genomics	The study aimed to develop a new gene selection method based on a fuzzy logic selection and classification algorithm, and to validate the gene signatures obtained on breast cancer patient cohorts	Breast cancer	Prognosis prediction	Fuzzy model	Rule-based system	Genomic	We confirmed the use of fuzzy logic selection as a new tool to identify gene signatures with good reliability and increased classification power	R package	NKI2-Agilent + KJX64KJ125-GSE2990 + Uppsala-GSE4922 + Transbig-GSE7390	Patients: 452

38	Yasser et al. [75], USA	BMC Medical Genomics	The aim was proposing a novel framework for multiomics data integration using multiview feature selection	Ovarian cancer	Predicting the clinical outcome and survival prediction	RF+ XGB +LR	Linear and nonlinear models	Genomic	Cancer survival prediction (short-term versus long-term survival)	Python	TCGA	Not mentioned
39	Chang et al. [76], South Korea	Scientific Reports	In this study, we have developed the Cancer Drug Response profile scan (CDRscan), a cancer genomic landscape-guided drug response prediction algorithm	More than one type of cancer	Treatment	CNN	Deep learning model	Drug response-functional data	Anticancer drug responsiveness	TensorFlow, Keras	CCLP + GDSC	Biosamples: Train set:144,953 Test set:7,641

40	Huang et al. [77], USA	Scientific Reports	The aim of this study was evaluating the performance of an approach to predict individual patient responses to drugs based on gene expression profiles of each individual's tumor	Ovarian cancer	Predict drug response	SVM	Linear model	Drug response-Functional data	Predicts individual cancer patient responses to chemotherapeutic drugs	Not mentioned	TCGA	Patients: 175

41	Shen et al. [28], China	IEEE Access	The aim was coming up with a new classification model named OFSSVM for cancer prediction using gene expression data	More than one type of cancer	Prognosis prediction	SVM	Linear model	Genomic	Multiclass cancer diagnosis	MATLAB	Prostate tumor dataset, AML/ALL dataset, GCM dataset	Not mentioned

42	Ow et al. [78], Singapore	Scientific Reports	The objective of this work was investigating the predictive performance of PSVM via optimization of the prognostic variable weights	Ovarian cancer	Prognosis prediction	SVM	Linear model	Genomic	Three survival-significant risk groups (low-, intermediate- or high- risk)	Python	TCGA + GSE9899 + GSE26712	Train: 349 patients Test: 359 patients
43	Xiao et al. [18], USA	Clinical Cancer Research	The aim was proposing a RWRF model, which updates the weight of each decision tree whenever additional patients` information is available, to account for the potential heterogeneity between training and testing data	Lung cancer	Predict drug response	RF	Linear and nonlinear models	Genomic	Predict the clinical response to gefitinib treatment	Not mentioned	Not mentioned	Test set: 59 patients genes: 1,473

44	Yu et al. [79], China	BMC Cancer	The aim of this study was providing a prediction model on the prognosis of lung adenocarcinomas based on somatic mutational features	Lung cancer	Prognosis prediction	SVM	Linear model	Genomic	Good and poor prognosis group	R package	TCGA	Patients:371

45	Dercle et al. [80], USA	European Journal of Radiology	Researchers aimed to develop a machine-learning algorithm for Quality Control of Contrast-Enhancement on CT-scan (CECTQC)	Liver cancer	Diagnosis + Treatment	RF	Linear model	Radiomics	Five contrast-enhancement phases in abdominal CT scan image	Python+ MATLAB+SPSS	Independent cohorts	Patients: 503

46	Zhang et al. [81], China	Radiotherapy and Oncology	A novel deep learning model was proposed to predict the risk for overall survival based on computed tomography images	Gastric cancer	Predict drug response	DL	Deep learning model	Radiomics	Risk prediction of overall survival	Not mentioned	Independent medical centers	Patients: 640

47	Su et al. [82], China	Methods	Researchers proposed a deep cascaded forest model, Deep-Resp-Forest, to classify the anticancer drug response as “sensitive” or “resistant”	More than one type of cancer	Predict drug response	Deep-Resp-Forest	Deep learning model	Genomic	Drug response prediction	Not mentioned	CCLE + GDSC	33 to 275 cancer cells lines + 12 to 156 cell lines
48	Mahmood et al. [83], South Korea	Journal of Personalized Medicine	Researchers proposed an AI-based nuclear segmentation technique which is empowered by residual skip connections to address this issue	More than one type of cancer	Prognosis prediction	CNN	Deep learning model	Genomic	Determination of cell phenotype, nuclear morphometrics, cell classification	MATLAB	TCGA + TNBC	Patients: 20,000

49	Malik et al. [84], India	BMC Genomics	The researchers proposed a multiomics integrative framework that robustly quantifies survival and drug response for breast cancer patients	Breast cancer	Prognosis prediction	NN	Deep learning model	Multiomics	Survival and drug response: two survival classes – high-risk and low risk	R package	TCGA+ GDSC	Patients: 6221

50	Nascimento et al. [85], Brazil	BMC Medical Informatics and Decision Making	A decision tree modeling was proposed to improve the accuracy of the pathogenicity identification process	More than one type of cancer	Treatment	DT	Nonlinear model	Genomic	Genetic variant impact prediction	Not mentioned	ClinVar	25.052 nonsynonymous mutations

51	Choi et al. [86], South Korea	Scientific reports	Researchers developed a novel Reference Drug-based Neural Network (RefDNN) model for effective prediction of anticancer drug response and identification of biomarkers contributing to drug resistance.	More than one type of cancer	Predict drug response	RefDNN	Deep learning model	Genomic + Drug Response-Functional data	Response to anticancer drugs	R package	GDSC + CCLE	1,065 cancer cell lines+ 983 cancer cell lines

52	Koras et al. [87], USA	Scientific Reports	Researchers compare standard, data-driven feature selection approaches to feature selection driven by prior knowledge of drug targets, target pathways, and gene expression signatures	More than one type of cancer	Predict drug response	LR+ RF	Linear and nonlinear models	Genomic + Drug Response-Functional data	Response to anticancer drugs	Python	GDSC	983 cancer cell lines
53	Zhu et al. [31], USA	Scientific Reports	Researchers investigate the power of transfer learning for three drug response prediction applications including drug repurposing, precision oncology, and new drug development	More than one type of cancer	Predict drug response	Elastic Net, RF, SVM	Linear and nonlinear models	Genomic + Drug Response-Functional data	Response to anticancer drugs	Not mentioned	GDSC+CCLE	1927 genes

54	Luo et al. [88], China	Pharmacological Research	Researchers propose a computationally efficient and cost-effective collaborative filtering method with ensemble learning to shorten the decision-making process regarding the selection of the most suitable compounds for patients	Lung cancer	Predict drug response	ECF-S + ECF-W	Linear and nonlinear models	Drug response-Functional data	Response to anticancer drugs	Not mentioned	Local dataset	Eight NSCLC (nonsmall cell lung cancer) cell lines

55	Maros et al. [30] Germany	Nature Protocols	Main aim was to perform a benchmark analysis to support the choice for optimal DNA methylation microarray data analysis through extensive comparisons of well-established ML classifiers such as RFs, ELNET, SVMs	More than one type of cancer	Treatment	RF+ ELNET + SVM	Linear and nonlinear models	Genomic	Classifying patients	R package + Python	TCGA	Patients: 2,801

56	Sharifi-Noghabi et al. [89], Canada	Bioinformatics	The main aim was predicting response to a drug given some single—or multiomics data	More than one type of cancer	Predict drug response	AITL	Deep learning model	Drug response-Functional data	Predict drug response	PyTorch	GDSC + TCGA+Patient-Derived Xenograft (PDX) Encyclopedia dataset+Patient datasets from nine clinical trial cohorts	Patient:618 targeted and chemotherapy drugs:299
57	Bazgir et al. [90], USA	Bioinformatics	The main aim of this study was anticancer drug sensitivity prediction using deep learning models for individual cell line	Not mentioned	Predict drug response	REFINED-CNN	Deep learning model	Drug response-Functional data	Anticancer drug sensitivity prediction	PaDEL	NCI60 + NCI-ALMANAC databases	17 cell lines

58	Woo et al. [91], Canada	Bioinformatics	The main aim is identification of a drug candidate causing a desired gene expression response, without utilizing any information on its interactions with protein target(s)	More than one type of cancer	Predict drug response	MLP	Deep learning model	Genomic + Drug Response-Functional data	Direct identification of a drug candidate causing a desired gene expression response	Python +R	LINCS CMap L1000 cancer genomic dataset	The gene profiles of 978 landmark genes

59	Jacobs et al. [92], USA	Cancers	The main aim of this study was identifying potential risk of local or systemic recurrence in breast cancer patients	Breast cancer	Prognosis prediction	SVM	Linear and nonlinear models	Radiomics	Classification of patients into different risk groups of breast cancer recurrence.	MATLAB	Johns Hopkins Integrated Breast Cancer Research Database	Patients: 80

60	Kaushik et al. [19], China	Chemical Biology and Drug Design	Main aim is predicting anticancer vaccine based on target sequence information using machine learning approach	More than one type of cancer	Predict vaccine response	NN	Linear and nonlinear models	Genomic	structure-based drug design	PERL programming language	Data from different resources	100 anticancer marks

61	Li et al. [93], China	10th Annual Computing and Communication Workshop and Conference (CCWC)	The main aim of this study was predicting the response of cell lines to drugs	More than one type of cancer	Predict drug response	CNN+LSTM	Deep learning model	Genomic + Drug Response-Functional data	Drug effectiveness prediction	PaDEL+TensorFlow	GDSC + COSMIC +TCGA	1074 cancer cell lines+ 17,419 genes in 1018 different cell lines+985 cancer cell lines
62	Laplante and Akhlouf [20], Canada	2020 42nd Annual International Conference of the IEEE Engineering in Medicine & Biology Society (EMBC)	The main aim was proposing a deep neural network classifier to identify the anatomical site of a tumor	More than one type of cancer	Cancer classification	NN	Deep learning model	Genomic	Discriminate between the different cancers	Not mentioned	TCGA	Not mentioned

63	Liu et al. [94], USA	Genes	Discover reliable and accurate molecular network-based biomarkers for monitoring cancer treatment	More than one type of cancer	Predict drug response	NBSBM	Bayesian model + Linear and non-linear models	Genomic	Predict drug response	Not mentioned	TCGA+ GSE17705+ GDSC	16 prostate cell lines+ 103 breast cancer patient+ 319 cancer cells

^∗^Abbreviation of AI methods defined by authors: SVM: support vector machine; RF: random forest; CNN: convolutional neural network; NB: naive Bayes; AF-UCS: attribute feedback-supervised classifier system; HNB: hidden Naive Bayes; MEFS: metaensemble feature selection; DT: decision trees; fuzzy logic selection algorithm MEMBA: membership margin based-attribute selection; NMTF: nonnegative matrix trifactorization; CDSS: clinical decision support system; KNN: K-nearest neighbor; NN: neural network; NLP: natural language processing; GBGFA: gene-wise prior Bayesian group factor analysis; OFSSVM: oriented feature selection SVM; KRL: Kernelized rank learning; PLATYPUS: Progressive LAbel Training bY Predicting Unlabeled Samples; LASSO: least absolute shrinkage and selection operator Cox regression; DenseNet: densely-connected classification network; WfG: Watson for Genomics; ENR: elastic net regression; C-T CERP: Classification-Tree CERP; CART: regression trees; MVL: multiview learning; LR: logistic regression; FE: feature extraction; PEMRIC: pharmacogenomics evidence mapping for reasoning with individualized clinical data; TCIA: The Cancer Immunome Atlas; GEO: Gene Expression Omnibus; CCLE: Cancer Cell Line Encyclopedia (CCLE); NSCLC: Nonsmall cell lung cancer treatment; CCLP: COSMIC cell line project; GDSC: Genomics of Drug Sensitivity in Cancer; CPTAC: Clinical Proteomic Tumor Analysis Consortium; CGP: comprehensive genomic profiling; NCICPD: Nursing CPD Institute; KRBDSR: Kent Ridge Biomedical Data Set Repository; DTI: drug-target interaction; EHR: electronic health record; PSPEP: Proteomics Performance Evaluation Pipeline Software; TCGA: The Cancer Genome Atlas; NACT: neoadjuvant chemotherapy; GRAPE: gene-ranking analysis of pathway expression; FNABs: fine needle aspirate biopsies; PSPEP: Proteomics Performance Evaluation Pipeline Software; EGFR: epidermal growth factor receptor; EGFR-TKI: EGFR tyrosine kinase inhibitors; RWRF: reweighted random forest; RPPA: reverse-phase protein array; BLCA: bladder urothelial carcinoma; ETE: extrathyroidal extension; PTC: papillary thyroid carcinoma; BAP1: BRCA1-associated protein; TR: tumor region; NCCH: National Cancer Center Hospital; XGB: eXtreme Gradient Boosting; AML: acute myeloid leukemia; ALL: acute lymphoblastic leukemia; PAAD: pancreatic ductal adenocarcinoma; LUAD: lung adenocarcinoma; PSVM: prognostic signature vector matching; MLP: multilayer perceptron; NDM: N-desmethyltamoxifen; ECF-S: ensemble collaborative filtering method with simple averaging; ECF-W: ensemble collaborative filtering method with weighted averaging; ELNET: elastic net; RefDNN: reference drug-based neural network; AITL: adversarial inductive transfer learning; NBSBM: network-based sparse Bayesian machine.

**Table 4:** Outcome measurements.

Reference	Effectiveness	Outcome
[45]	The mean of mPS = 24.22 (interquartile range [IQR] of 15.56–33.60).	(i) The MPS system is simple and cost-effective to apply and yet can reveal previously unrecognized heterogeneity among patient subpopulations in a platform-independent manner.

[64]	Radiomics model: AUC = 0.956, specificity = 0.928, sensitivity = 0.896. transcriptomics model: AUC specificity and sensitivity 0.948, 1, and 0.676.	(i) The integrative nomogram incorporated CECT radiomics, transcriptomics, and clinical features improved the PFI prediction in BLCA patients and is a feasible and practical reference for oncological precision medicine.

[27]	AUC, 0.837, *p* < 0.001; F1 score, 0.766.	(i) The radiomics signature model achieved a better classification performance than radiologists, which demonstrated the impressive prediction ability of radiomics signature.

[79]	Accuracy of 81% and AUC of 0.896 for the ROC curves.	(i) The model exhibited good interstage prognosis prediction performance. The genetic features could be used as biomarkers for effective LUAD prognosis prediction

[51]	Accuracy: decision tree:70.8% Discriminant analysis (Linear): 66.9% Linear SVM: 69.6% Weighted KNN: 73.5% ensemble classified (Subspace discriminant): 70.0%.	(i) The proposed methods were able to distinguish the metastatic sclerotic lesions with a complete response.

[70]	Accuracy = 0.9143.	(i) This synergy between liquid biopsy biotechnology and XAI will surely lead to personalized interpretable medicine, ensuring adequate and better diagnostic tools and treatments.

[60]	AUC = 0.97 − 0.98.	(i) A PLATYPUS model trained on the drug trial data can predict drug response for this patient without retraining.

[69]	Sensitivity = 97.1%, specificity = 98.8%, ROC curves = 0.99.	(i) This was concluded that this deep learning model provides an accurate and reproducible method for the prediction of BAP1 expression in uveal melanoma.

[25]	Sensitivity: upper than 84% in the training set but below 77% in the testing set.	(i) This study demonstrated that MRI-based radiomics features hold potential in the pretreatment prediction of response to NACT in LACC, which could be used to identify rightful patients for receiving NACT avoiding unnecessary treatment.

[59]	The specificity, sensitivity and accuracy respectively: 0.861, 0.641, and 0.747.	(i) The TCPR model may benefit decision-making regarding total laryngectomy or larynx-preserving treatment. This TCPR model incorporating radiomics signature and T category reported by radiologists has good potential to be applied for individual accurate preoperative T category.

[63]	(MAE) 4.112E-06, (MSE) 4.318E-06.	(i) The proposed framework had demonstrated its capability and potential for mapping the gene and tumor status, it was effective for detecting association between gen information and the tumor growth regions.

[76]	AUC = 0.98.	(i) CDRscan is expected to allow the selection of the most effective anticancer drugs for the genomic profile of the individual patient.

[43]	The average correlation coefficient: 0.438-0.461.	(i) The result shows that GloNetDRP achieves comparable performance on the two-omics data for eight drugs collected from CCLE and GDSC. GloNetDRP globally calculated the responses of untested cell lines for the query drug by considering not only the neighbors but also other drugs and cell lines.
[26]	Precision = 0.98, recall = 0.99, and F − score = 0.98.	(i) Clinical or pathology notes alone or together provided the broadest cohort coverage and clinical notes alone provided the most precise measure of receptor status.

[58]	Concordance rate = 94.5% (95% CI, 92.7–96.0%) for gene mutations.	(i) WfG showed comparable analytical results for clinical genome sequencing. WfG demonstrated a significant improvement in mutation assignment from ver. 27 and 33. WfG may be useful in cases where large amounts of genomic data are available

[55]	Docetaxel and bortezomib with AUROCs of 0.74 and 0.71, respectively.	(i) The proposed was approach outperforms several state-of-the-art predictors in drug recommendation, if the training dataset is sparse, and generalizes to patient data.

[77]	Study1: the overall accuracies GEM 81.5%; 5-FU 81.7%; study 2: overall accuracy: 82.6%.	(i) ML-based models with validated high positive predictive values may provide physicians with a useful alternative to the traditional trial-and-error strategies.

[75]	AUC scores of RF: 0.66 XGB: 0.66 LR: 0.66 MV: 0.7	(i) Our results demonstrate the potential of multiview feature selection in integrative analyses and predictive modeling from multiomics data.

[28]	Accuracy (%) 97.06 AUC = 0.9929	(i) The experiments show that the OFSSVM is an appealing compromise between interpretability and classification accuracy, and is superior to other traditional methods in the sense of comprehensive evaluation.

[72]	FPR for DNT and DMT *p* values at *α* = .05 for Sc1: 0.04 and 0.208	(i) PANOPLY can be a tool to help clinicians in their decision-making process.

[56]	SCNN models median c index 0.745, *p* = 0.307 GSCNN models: 0.754 to 0.801.	(i) The proposed approach surpasses the prognostic accuracy of human experts for classifying brain tumors.

[44]	ROC curve of the Gemelli polyclinic's data set = 0.759. ROC curve of the Maastricht clinic = 0.881. ROC for the testing set was depicted 0.603 and 0.588 for each data set.	(i) Experimental results indicate that the system can generate a highly performant center-specific predictive model.

[73]	Accuracy across all pathways was 0.96 for a single dataset and 0.72 with multiple datasets	(i) GRAPE pathway scores provide researchers with a unique perspective of patient transcription profiles that may lead to improvements in the prediction performances of a wide range of personalized medicine applications.

[54]	For CTRP panel, the median was calculated for GBGFA, ENET 0.05, and 0.04. For CCLE panel, the median was calculated for GBGFA and ENET 0.06, 0.02.	(i) Current results show that the GBGFA model enables leveraging information from combinations of genomic data which improves the predictive performance and feature selection as compared to Elastic Net and BGFA.

[48]	Sensitivity = 0.82 and specificity = 0.82.	(i) The results suggested the effective therapies for the majority of cancer cells investigated in the dataset.

[57]	Recall, precision, and F2: 0.39, 0.21, and 0.33.	(i) This QA system can be effective for helping physicians in relevant knowledge. So, precision oncology can provide fewer toxic treatments in neoplasms.

[78]	More than 90% accuracy	(i) The analysis demonstrated that voting of the output categorical values for a given patient across distinct prognostic/classification methods could lead to a more robust, accurate, reproducible, and cost-efficient prognostic/ classification strategy for precision medicine.

[52]	The FNR and FPR values: 0.0512, 0.037.	(i) The proposed algorithm improves the cost efficiency and accuracy of the screening process compared to current clinical practice guidelines.
[50]	The best area under the ROC = 0.80 and the best PR (precision − recall) curve = 0.83.	(i) The proposed approach has the potential to enable the derivation of new hypotheses, improve drug selection, and lead to an improvement in patient genomics-tailored therapeutics for cancer.

[71]	Range of AUC 0.58-0.64	(i) Such studies are expected to contribute to precision medicine and better guide treatment for these deadly diseases

[74]	(≥0.80 % accuracy for 10 drugs, ≥75 % accuracy for 19 drugs	(i) This model could be applied to predict drug response for some certain drugs and potentially play a complementary role in personalized medicine.

[29]	Sensitivity from 90% to 95%, specificity 67% to 93%).	(i) This study opens the way to further development for identification of new biomarker combinations in other applications such as prediction of treatment response.

[49]	Sensitivity = 43.8%, specificity = 100%, identical to qPCR on the same samples.	(i) The ability of Afirma BRAF to accurately detect V600E status may assist physicians in making these treatment decisions and potentially improve patient care.

[68]	Sensitivity = 96.77% on the training set. The model achieved 91. Specificity = 91.67%sensitivity = 100%. Test cases.	(i) In this study, the CANScript platform was versatile in its ability and capacity to predict the outcomes of both cytotoxic chemotherapy regimens and targeted therapeutics.

[95]	The accuracy of SMO, J48, RF, and CART was calculated respectively 76.56%, 75%, 75%, and 73%.	(i) The findings suggested that decision trees and support vector machines are engaged approaches for clinical decision support in the patient selection for targeted therapy in advanced NSCLC.

[53]	Accuracy = 0.99, sensitivity = 0.98, Jaccard index (stability) = 0.80	(i) The results have shown that MEFS improve the robustness and the accuracy of the signature and outperforms other methods in the literature

[18]	Accuracy = 0.84	(i) The proposed RWRF model can improve the prediction accuracy significantly. The method can facilitate using molecular signatures to predict the clinical outcomes of patients in prospective clinical studies.

[67]	Average accuracy for leukemia: 92.90%; breast 84.67%; colon cancer 86.53%;	(i) The proposed two-step Bayes classification framework was equal to and, in some cases, outperformed other classification methods in terms of prediction accuracy, the minimum number of classification markers, and computational time.

[65]	Pearson correlation Rp = 0.85; coefficient of determination R2 = 0.72, RMSE = 0.83	(i) This model had been shown that the prediction of drug response and mode of action by transcriptional profiling is significantly and effectively enhanced when paired with known a priori gene and protein networks.

[47]	The average training accuracy of 0.6995 and average testing accuracy of 0.6042.	(i) This investigation implicated XPD 751, XPD 312, and pack-years of smoking as significant predictors of bladder cancer susceptibility.

[46]	This algorithm performed better than simple metrics for variation in individual and multiple genes (*R*^2^ = 0.10; *p* < 0.05).	(i) This approach performed better than simple metrics for variation in individual and multiple genes

[62]	Average accuracy for SVM, NBC, and BCN was calculated respectively 85.7, 94.2, and 94.3.	(i) As this contribution, the experiments with lung cancer data prove that RPPA data can be used to profile patients for drug sensitivity prediction.

[61]	The confident predictability (CP), error in CP, the total error was respectively 98%, 4.23%, and 4.17 %, for the GCM dataset.	(i) The author believed that this method can be a useful tool for translating the gene expression signatures into clinical practice for personalized medicine.

[66]	Accuracy, sensitivity, specificity, PPV and NPV of respectively 0.872, 0.846, 0.882, 0.851 and 0.89.	(i) The C-T CERP algorithm appears to have a good potential and effective role for biomedical decision making in the assignment of patients to treatment therapies.
[80]	The CECT-QC algorithm reached an overall accuracy of 79.4% [95%CI = 75.2%, 82.9%].	(i) This study demonstrated that the CECT-QC algorithm is useful for radiomic-based precision diagnosis

[81]	The trained DL model classified patients into high-risk and low-risk groups in training cohort (*p* value < 0.001, concordance index (C-index): 0.82, hazard ratio (HR): 9.79) and external validation cohort (*p* value < 0.001, C-index: 0.78, HR: 11.76).	(i) DL model can provide CT-based prognostic risk scores related to the OS of GC patients, and the findings demonstrated higher prognostic value than clinical and radiomics models.

[82]	Average accuracy of 85% and AUC is 93%.	(i) The results show quite a high prediction accuracy, which proves the discriminative ability of the proposed model.

[83]	F1-measure of 0.8547 on TCGA dataset, precision of 0.8352, recall of 0.8306, and F1-measure of 0.8329 on the TNBC dataset.	(i) The proposed R-SNN maintains crucial features by using the residual connectivity from the encoder to the decoder, and it also uses only a few layers, which reduces the computational cost of the model.

[84]	The accuracies of training, validation, and test dataset were 93.5, 93.7 and 98.1%, respectively; AUROC value of 0.98 was observed for both the classes.	(i) The proposed omics integration strategy provides an effective way of extracting critical information from diverse omics data types enabling estimation of prognostic indicators.

[85]	The highest precision: 91% for true neutrals, 8% for false neutrals, 9% for false pathogenic, and 92% for true pathogenic.	(i) The decision tree exceptionally demonstrated high classification precision with cancer data, producing consistently relevant forecasts for the sample tests with an accuracy close to the best ones achieved from supervised ML algorithms.

[86]	On the GDSC dataset, the AUCROC of RefDNN were 0.891; the AUCROC of RefDNN were 0.071 on the CCLE dataset.	(i) As the proposed model can guarantee good prediction of drug responses to untrained drugs for given gene expression patterns, it may be of potential benefit in drug repositioning and personalized medicine

[87]	The median AUC value per target pathway ranges from 0.98 for hormone-related drugs to 0.73 for compounds targeting metabolism pathways.	(i) Appropriate feature selection strategies facilitate the development of interpretable models that are indicative for therapy design.

[31]	AUC value 0.98 and 0.99.	(i) The results demonstrate proposed framework improves the prediction performance in all three drug response prediction applications with all three prediction algorithms.

[88]	Average accuracy of ECF-W and ECF-S is 74.25% and 77.25%, respectively.	(i) These two methods recommend the most suitable compounds and anticancer drugs for patients with NSCLC.

[30]	The highest AUC of RF, ELNET and SVM are 99.9%, 99.8%, and 85.0%, respectively.	(i) The protocols developed as a result of these comparisons provide valuable guidance on choosing ML workflows and their tuning to generate well-calibrated CP estimates for precision diagnostics using DNA methylation data.

[89]	The highest AUROC: 0.74	(i) The empirical results indicated that AITL achieved a significantly better performance compared with the baselines showing the benefits of addressing the discrepancies in both the input and output spaces.

[90]	The highest performance: 0.71	(i) REFINED-CNN improves the prediction performance as compared to the best single REFINED CNN model.

[91]	The highest performance: 0.84	(i) This method did not show ideal results when applied to an external set but it provided a valid proof of principle starting point, termed for future improvement.

[92]	Sensitivity: 95% Specificity: 83% AUC: 0.89	(i) This method provided more quantitative metrics for better characterization and complete picture of breast lesions.
[19]	Precision: 95%	(i) It might be very useful in new target recognition as well and proposing a potent drug for the newly identified target

[93]	The highest R2: 0.84	(i) This model provides a new method for the prediction of anticancer drugs in human tissues and can provide some reference value for the screening of anticancer drugs.

[20]	Accuracy: 96.9%	(i) Their results demonstrate the possibility of using stem-loop expression data for accurate cancer localization.

[94]	The highest AUC: 0.942	(i) The results showed that the proposed algorithm performed much better than the other two methods, warranting further studies in individual cancer patients to predict personalized cancer treatments.

**Table 5:** Distribution of applied AI algorithms and their categorizations by frequencies.

Row Labels	Frequency
*Linear and nonlinear models*	17
DT+ SVM+ KNN+EC	1
ECF-S + ECF-W	1
Elastic Net+ RF+ SVM	1
KRL	2
LR+ RF	1
LR+ RF+ SVM	1
MLP + RF	1
NN	1
RF	2
RF + SVM	1
RF+ ELNET + SVMs	1
RF+ NN	1
RF+ XGB +LR	1
SVM	1
SVM + DT	1
*Deep learning model*	15
AITL	1
CDSS	1
CNN	5
CNN+LSTM	1
Deep-Resp-Forest	1
DenseNet-121	1
DL	1
MLP	1
NN	3
*Linear model*	15
CART	1
GloNetDRP	1
MEFS	1
NMTF	1
RF	1
SVM	10
*Rule-based system*	6
CDSS	6
*Bayesian model*	3
GBGFA	1
NB	1
NB + HNB	1
*Nonlinear model*	3
AF-UCS	1
DT	1
LASSO	1
*Bayesian model + Linear and nonlinear models*	2
NB+ BM	1
RF+ SVM+NB	1
*NLP*	2
NLP	2
Grand Total	63

## Data Availability

All data generated or analyzed during this study are included in this published article.
